# Supplementary material for: Serum from COVID-19 patients early in the pandemic shows limited evidence of cross-neutralization against variants of concern
Source: Sci Rep. 2022 Mar 10;12:3954. doi: 10.1038/s41598-022-07960-4 (PMC8913826; doi:10.1038/s41598-022-07960-4)
Supplement: Supplementary file 1 — Supplementary Information. [file 41598_2022_7960_MOESM1_ESM.pdf]

Serum from COVID-19 patients early in the pandemic shows limited evidence of cross-neutralization against variants of concern

Amanda J. Griffin[1], Kyle L. O'Donnell[1], Kyle Shifflett[1], John-Paul Lavik[2], Patrick M. Russell[2], Michelle K. Zimmerman[2], Ryan F. Relich[2], and Andrea Marzi[1]*

[1]Laboratory of Virology, Division of Intramural Research, National Institute of Allergy and Infectious Diseases, National Institutes of Health, Hamilton, MT 59840, USA

[2]Department of Pathology and Laboratory Medicine, Indiana University School of Medicine, Indianapolis, IN 46202, USA

Supplementary materials

Supplementary Figures 1-4

Supplementary Table 1

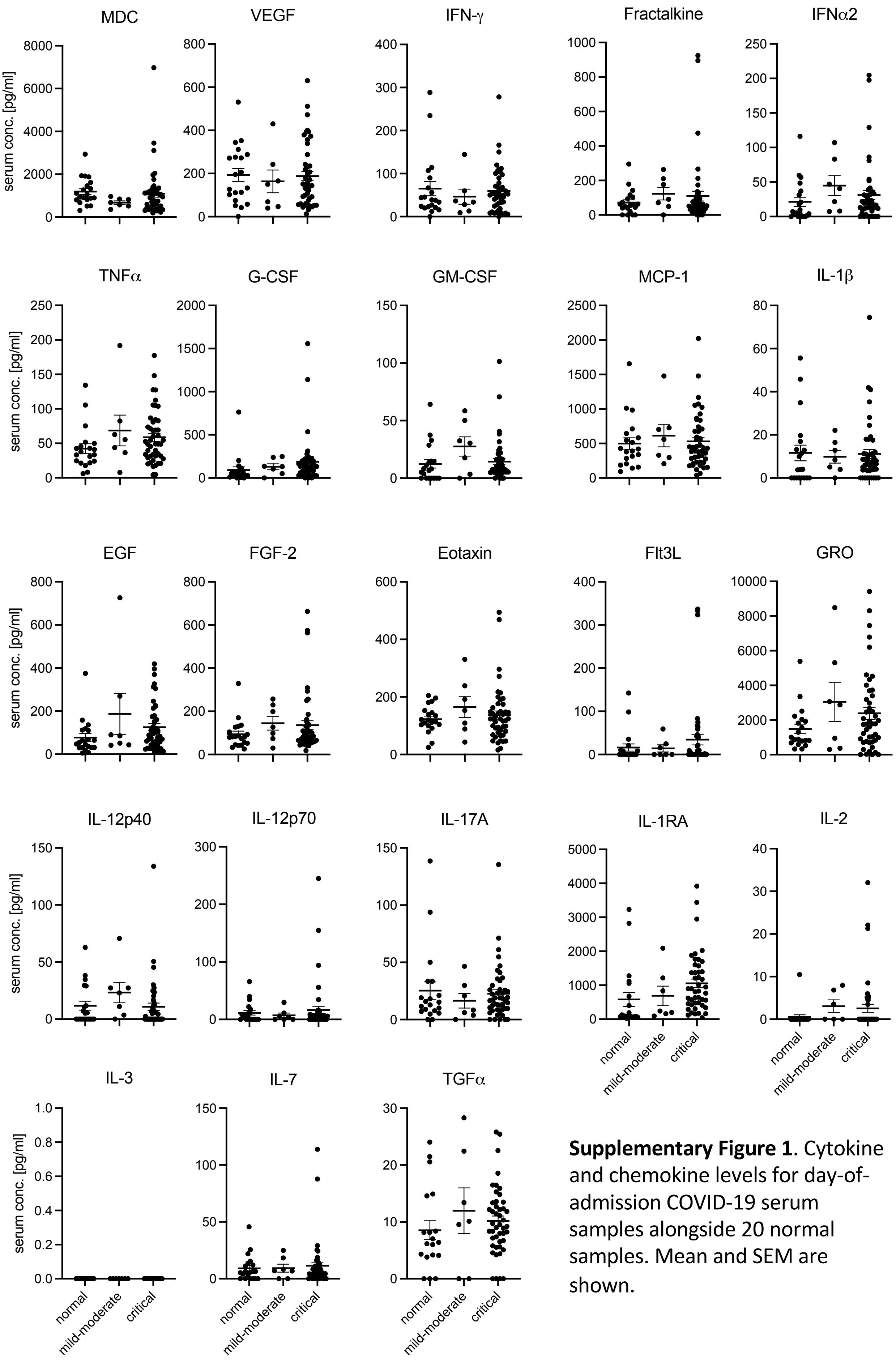

Supplementary Figure 1. Cytokine and chemokine levels for day-of-admission COVID-19 serum samples alongside 20 normal samples. Mean and SEM are shown.

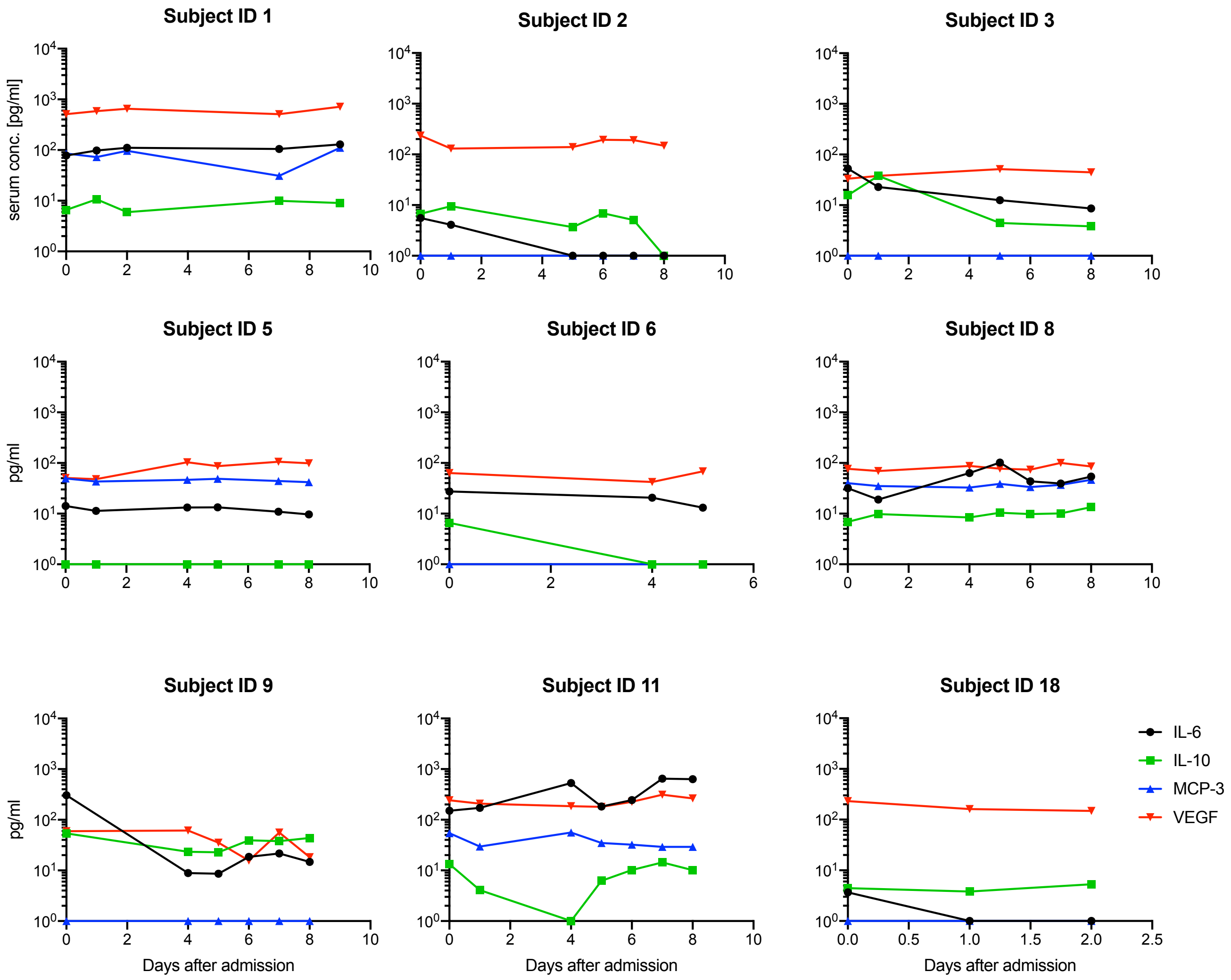

Supplementary Figure 2. Cytokine and chemokine levels of selected patients over time.

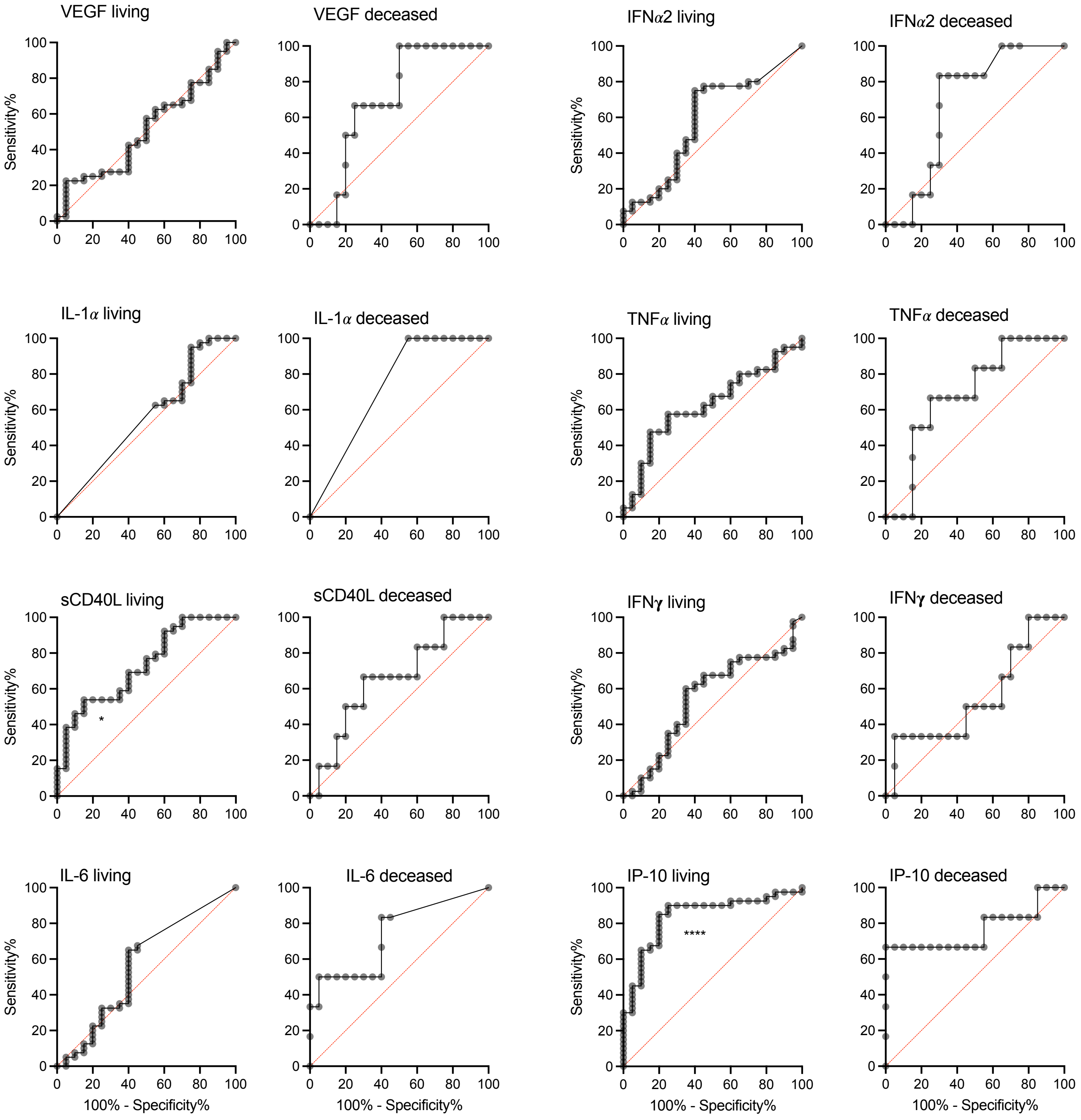

Supplementary Figure 3. ROC curves were calculated for analytes shown in Fig. 2 comparing "normal vs. living" or "normal vs. deceased". Statistically significant differences for sCD40L and IP-10 in the "living" group are indicated as $p<0.0001$ (****), and $p<0.05$ (*).

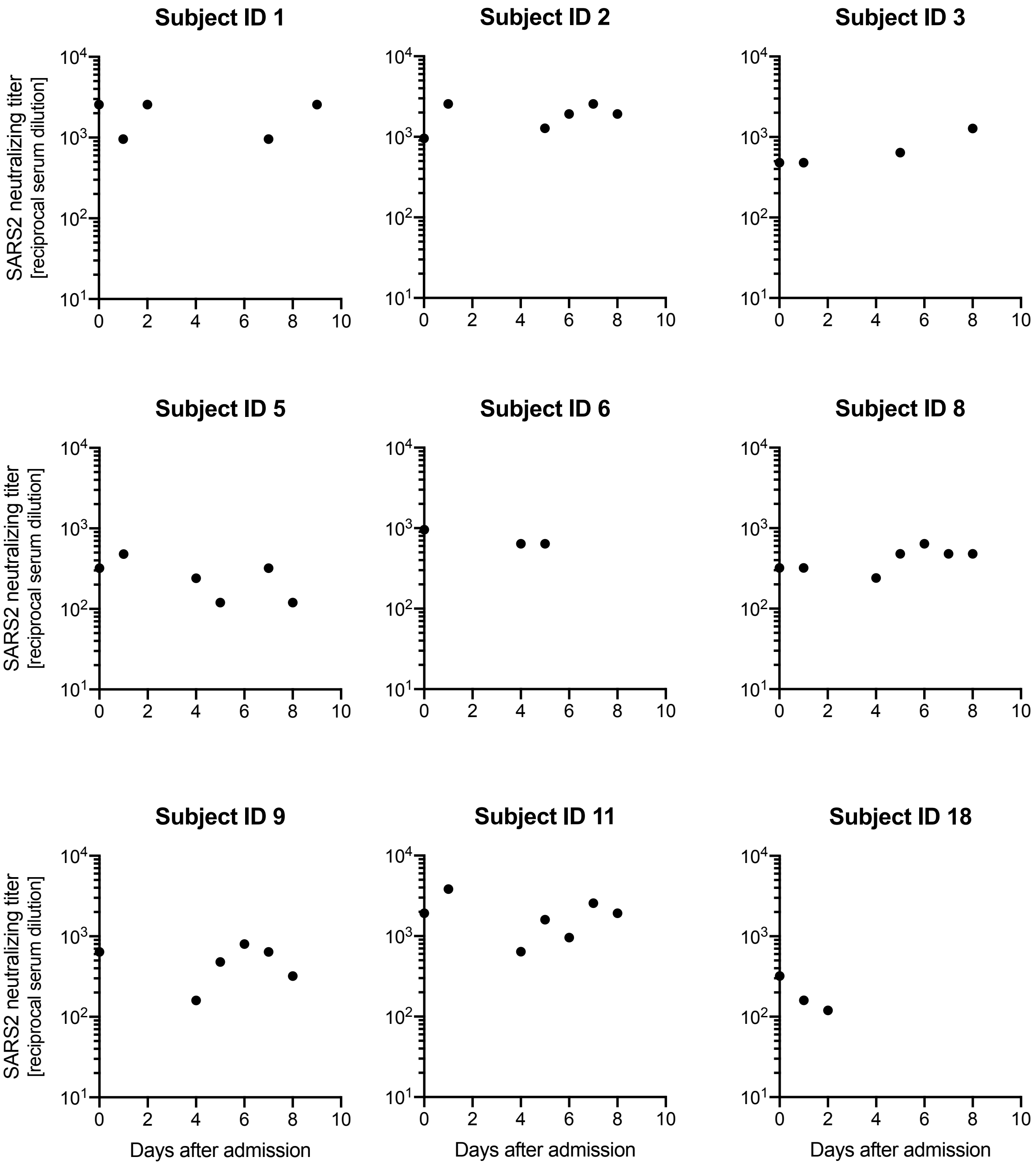

Supplementary Figure 4. Anti-SARS-CoV-2 neutralizing antibodies remain steady over time in selected patients.

	normal vs. living		normal vs. deceased		living vs. deceased	
	n=20	n=40	n=20	n=6	n=40	n=6
	**AUC**	***p***	**AUC**	***p***	**AUC**	***p***
**VEGF**	0.51	0.9002	0.7	0.1441	0.6875	0.1422
**IFNα2**	0.5863	0.2792	0.6833	0.1806	0.6083	0.3964
**IL-1α**	0.5519	0.5152	0.725	0.1003	0.6875	0.1422
**TNFα**	0.6263	0.1132	0.6917	0.1616	0.5125	0.9221
**sCD40L**	0.7282	0.0044	0.6583	0.2476	0.6325	0.3006
**IFNγ**	0.5481	0.546	0.55	0.715	0.6	0.4337
**IL-6**	0.5669	0.4015	0.7375	0.0828	0.6896	0.1378
**IP-10**	0.84	<0.0001	0.7667	0.0515	0.6458	0.2536

Supplementary Table 1. Area under the curve (AUC) from ROC analysis of select cytokines. Statistically significant differences are highlighted in red font.
